# Insights into the Action Mechanism of the Antimicrobial Peptide Lasioglossin III

**DOI:** 10.3390/ijms22062857

**Published:** 2021-03-11

**Authors:** Filomena Battista, Rosario Oliva, Pompea Del Vecchio, Roland Winter, Luigi Petraccone

**Affiliations:** 1 Department of Chemical Sciences, University of Naples Federico II, Via Cintia, 4, 80126 Naples, Italy; filomena.battista@unina.it (F.B.); pompea.delvecchio@unina.it (P.D.V.); 2 Faculty of Chemistry and Chemical Biology, Physical Chemistry I, TU Dortmund University, Otto-Hahn-Str. 4a, 44227 Dortmund, Germany; rosario.oliva@tu-dortmund.de (R.O.); roland.winter@tu-dortmund.de (R.W.)

**Keywords:** Lasioglossin LL-III, antimicrobial peptides, liposomes, calorimetry, fluorescence, circular dichroism, leakage assay, lipid domains

## Abstract

Lasioglossin III (LL-III) is a cationic antimicrobial peptide derived from the venom of the eusocial bee *Lasioglossum laticeps*. LL-III is extremely toxic to both Gram-positive and Gram-negative bacteria, and it exhibits antifungal as well as antitumor activity. Moreover, it shows low hemolytic activity, and it has almost no toxic effects on eukaryotic cells. However, the molecular basis of the LL-III mechanism of action is still unclear. In this study, we characterized by means of calorimetric (DSC) and spectroscopic (CD, fluorescence) techniques its interaction with liposomes composed of a mixture of 1-palmitoyl-2-oleoyl-*sn*-glycero-3-phosphocholine (POPC) and 1-palmitoyl-2-oleoyl-*sn*-glycero-3-*rac*-phosphoglycerol (POPG) lipids as a model of the negatively charged membrane of pathogens. For comparison, the interaction of LL-III with the uncharged POPC liposomes was also studied. Our data showed that LL-III preferentially interacted with anionic lipids in the POPC/POPG liposomes and induces the formation of lipid domains. Furthermore, the leakage experiments showed that the peptide could permeabilize the membrane. Interestingly, our DSC results showed that the peptide-membrane interaction occurs in a non-disruptive manner, indicating an intracellular targeting mode of action for this peptide. Consistent with this hypothesis, our gel-retardation assay experiments showed that LL-III could interact with plasmid DNA, suggesting a possible intracellular target.

## 1. Introduction

In the era of antibiotics, one of the powerful approaches to developing novel antimicrobial agents is to borrow from the wisdom of nature [1]. Natural antimicrobial peptides (AMPs) were first characterized in the 1980s [2]; since then, AMPs have been discovered in diverse species of fungi, plants, and animals [3,4]. They comprise a wide range of short, cationic peptides that constitute the first line of innate immune defense against infectious agents [5,6]. To date, more than 3100 AMPs have been identified and registered into the updated antimicrobial peptide database APD3 [7]. Although the precise mechanism of AMPs is still not known, it is generally accepted that these positively charged peptides target the membrane of pathogens [8,9,10]. In most cases, the function of the membrane is irreversibly disturbed, leading to the loss of cellular contents and rapid cellular death [11]. In addition, some AMPs flip from the outer to the inner face of the plasma membrane and then into the cytoplasm, where they find an intracellular target [12]. Lasioglossins, a new family of antimicrobial peptides, were recently discovered in the venom of the eusocial bee *Lasioglossum laticeps*. Three structurally related lasioglossins have been identified and named Lasioglossin I, II, and III [13]. They showed strong antimicrobial activity against both Gram-positive and Gram-negative bacteria with low hemolytic activity against rat erythrocytes and low induction of mast cell degranulation [13,14,15]. Of the three peptides, Lasioglossin III (LL-III) showed the best antimicrobial activity. In addition, LL-III showed antitumor as well as antifungal properties [13,16,17]. Moreover, recently it was demonstrated that LL-III could also perturb coacervates composed of LAF-1 and RNA [18]. Due to its large range of antimicrobial activity, LL-III seems to be a very good lead compound in the development of new drugs for human therapy. However, the exact mechanism through which its activity is carried out is not completely understood. It was reported that LL-III could target some intracellular components in cancer cells [16]. However, how the peptide can reach the intracellular milieu is not clear. Furthermore, there is no report on the detailed interaction of LL-III with membrane mimetic liposomes to date, which is important to reveal the specific role played by the lipid-peptide interactions in the action mode of LL-III. To elucidate the key features of the underlying action mechanism, we performed a detailed biophysical characterization of the interaction of LL-III with 1-palmitoyl-2-oleoyl-*sn*-glycero-3-phosphocholine (POPC)/1-palmitoyl-2-oleoyl-*sn*-glycero-3-*rac*-phosphoglycerol (POPG) liposomes as a model of pathogenic anionic membranes. The choice of these lipids was based on the observation that the membrane composition of bacterial (prokaryotic) and cancer (eukaryotic) cells, although different, share a common feature: the presence of negatively charged lipids in the outer leaflet of the bilayer [19,20,21,22]. In addition, to rationalize the low toxic effect against healthy eukaryotic cells, model membranes composed of only POPC were also employed. Our data show that the peptide binds to both model membranes but with a slightly higher affinity for the negatively charged POPC/POPG liposomes in which it induces lipid segregation. Furthermore, experimental leakage results show that the peptide is able to permeabilize the membranes. Interestingly, our DSC results show that the peptide-membrane interaction occurs in a non-disruptive way, pointing toward an intracellular targeting mode of action for this peptide. Consistently with this hypothesis, our gel-retardation assay experiments show that LL-III can interact with plasmid DNA, suggesting a possible intracellular target.

## 2. Results

### 2.1. Binding of LL-III to Model Membranes

**Figure 1:**
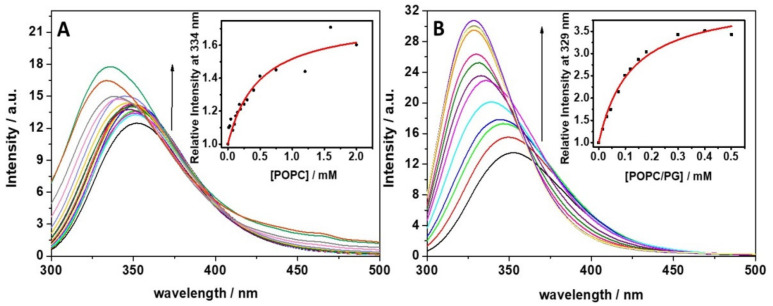
Fluorescence emission spectra of the Lasioglossin III (LL-III) peptide at increasing concentrations of (**A**) 1-palmitoyl-2-oleoyl-*sn*-glycero-3-phosphocholine (POPC) and (**B**) POPC/1-palmitoyl-2-oleoyl-*sn*-glycero-3-*rac*-phosphoglycerol (POPG) large unilamellar vesicles (LUVs) obtained upon excitation at 280 nm. Black arrows indicate the increasing LUVs concentration. The experiments were performed in 10 mM phosphate buffer, pH 7.4, at 25 °C. The insets show the binding isotherms. The red lines represent the best fit of experimental data.

In studying peptide-membrane interactions, it is of fundamental importance to verify and quantify the propensity of a peptide to bind to model membranes. To this end, we followed the fluorescence intensity changes of the Trp residue of the LL-III upon the addition of lipid vesicles. In Figure 1, fluorescence emission spectra of the LL-III peptide with the increasing concentration of POPC (Panel A) and POPC/POPG (Panel B) large unilamellar vesicles (LUVs) are shown. Upon addition of POPC vesicles up to 2 mM, a modest but detectable increase in the emission intensity is observed (the fluorescence emission increases 0.3 times with respect to the free peptide). The increase in the intensity is accompanied by a clear blue shift of the maximum of emission (~16 nm), revealing that the Trp residue was experiencing a more hydrophobic environment. Similarly, upon the addition of POPC/POPG vesicles (up to 0.5 mM), a fluorescence emission increase and a blue shift of the maximum were observed. However, in this case, both the fluorescence enhancement and the blue shift (~23 nm) were much stronger compared to POPC and occurred in a smaller lipid concentration range. All these results reveal that the peptide is able to interact with both model membranes, although with different affinity and/or with different binding modes. To quantitatively compare the LL-III affinity for the two membranes, we determined the mole fraction partition constants (*K*_x_) by fitting the corresponding binding isotherms (insets of Figure 1). The obtained values were 1.1 ± 0.7 × 10^5^ and 4.5 ± 1.0 × 10^5^ for POPC and POPC/POPG, respectively, revealing that LL-III had a slightly higher affinity for the negatively charged model membrane.

### 2.2. Conformational Changes of LL-III upon Binding

**Figure 2:**
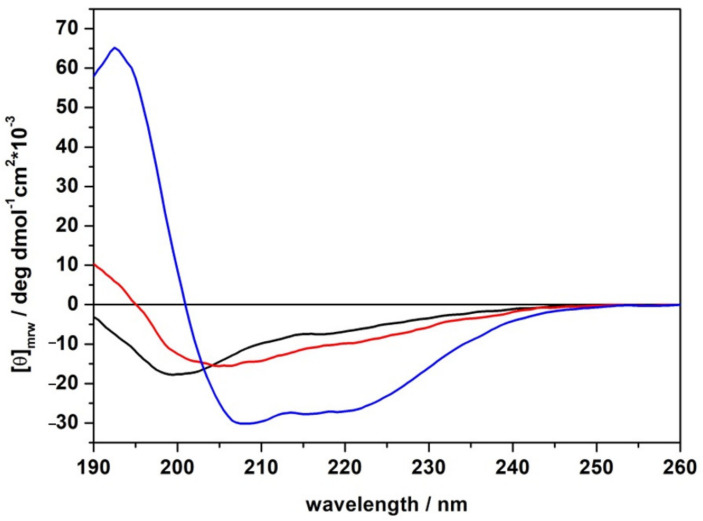
Far-UV circular dichroism (CD) spectra of the LL-III peptide in neat buffer (black line), in the presence of POPC (red line) and POPC/POPG (blue line) at a lipid-to-peptide ratio of 50. The spectra were recorded in 10 mM phosphate buffer, pH 7.4, at the temperature of 25 °C.

Circular dichroism (CD) spectroscopy experiments were carried out in order to assess the conformation of the LL-III peptide upon membrane interaction. In Figure 2, Far-UV spectra of LL-III peptide in buffer solution and in the presence of POPC or POPC/POPG LUVs are shown. To quantitatively evaluate the secondary structure content, we performed a deconvolution of the spectra by means of PEPFIT software [23,24,25]. The results are reported in Table 1.

The CD spectrum of LL-III in the absence of vesicles showed a negative band at about 200 nm, indicating that the peptide was mainly not structured in solution. However, small fractions of α-helix and β-turn were inferred from our spectral deconvolution, in agreement with previously reported data [13]. After the addition of POPC vesicles, the CD spectrum slightly changed, showing a negative band at around 205 nm and a weak positive band at 190 nm, indicating that the peptide is more structured with respect to the neat buffer conditions. The deconvolution analysis reveals a small increase in helix content and a decrease in random coil structure. On the contrary, the peptide’s CD spectrum drastically changed upon the addition of POPC/POPG vesicles. Particularly, the spectrum showed two well-defined minimums at around 208 nm and 220 nm and a maximum at 195 nm, revealing that the LL-III adopted a helical structure. Consistently, the deconvolution analysis reveals a strong increase in the α-helix content (from 15% to 89%) upon the addition of POPC/POPG.

### 2.3. The Effects of LL-III on the Membranes’ Microstructure and Thermotropic Properties

**Figure 3:**
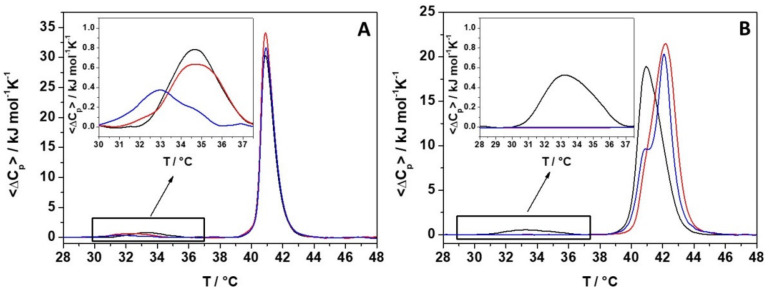
DSC profiles of 1,2-dipalmitoyl-*sn*-glycero-3-phosphocholine (DPPC) (**A**) and DPPC/ DPPC/1,2-dipalmitoyl-*sn*-glycero-3-phosphoglycerol (DPPG) (**B**) multilamellar vesicles (MLVs) (black lines) at L/P of 50 (red lines) and 10 (blue lines). The insets show an enlargement of the pre-transition peaks.

We then investigated the effects of peptide binding to the model membranes on the thermotropic properties of the membranes by means of differential scanning calorimetry (DSC). To this end, POPC and POPC/POPG vesicles were replaced with 1,2-dipalmitoyl-*sn*-glycero-3-phosphocholine (DPPC) and DPPC/1,2-dipalmitoyl-*sn*-glycero-3-phosphoglycerol (DPPG) vesicles. These replacements were needed since the POPC and POPG exhibit a transition temperature below zero, not suitable for DSC measurements [23,26]. In Figure 3, DSC thermograms of DPPC (panel A) and DPPC/DPPG (panel B) at different lipid-to-peptide (L/P) ratios are shown, and the corresponding thermodynamic parameters are shown in Table 2.

DSC thermograms of the DPPC and DPPC/DPPG vesicles in the absence of peptide were very similar and characterized by two transitions, at about 33 °C and 41 °C, respectively, termed pre-transition and main transition [27,28]. The pre-transition peak represents the transition from the lamellar gel phase (L_β′_) to the rippled gel phase (P_β′_), and it is mainly due to the rearrangement of lipid head groups. Instead, the main transition peak is the transition from the rippled gel phase (P_β′_) to the liquid crystalline phase (L_α_). It is due to the melting of lipid acyl chains which pass from an all-trans to gauche conformations. Thus, by looking at the two transitions, it was possible to monitor the effect of peptide interaction on two distinct regions of the membrane, the membrane surface and the hydrophobic core.

For DPPC, the addition of the peptide significantly affected the pre-transition peak, lowering both T_p_ and ΔH_p_ (Table 2). Conversely, the presence of the peptide had no effect on the main transition, suggesting that the peptide mainly perturbs the lipid head group region rather than the lipid tail region.

For the DPPC/DPPG model membrane, increasing the peptide concentration drastically affected the pre-transition, leading to a complete disappearance of the peak at L/P = 50, indicating a strong interaction with the lipid head groups on the membrane surface. Unlike the DPPC vesicles, the presence of the LL-III peptide has a marked effect also on the main transition of DPPC/DPPG vesicles. At L/P = 100, the peak shape changed, and the transition peak shifted at a higher temperature. At L/P = 50, the DSC peak was asymmetric, with a small shoulder around 40.8 °C. At L/P = 10, the thermogram shows two well distinct peaks centered at 41 °C and 42 °C. These temperatures were surprisingly very similar to the melting temperature of pure DPPG and DPPC lipids and suggest that the peptide binding could partially induce lipid segregation. Interestingly, even if the peptide was able to dramatically affect the shape of the gel-to-liquid transition, the overall enthalpy changes of the gel-to-liquid phase transition were not significantly affected by the presence of the peptide (Table 2). This observation indicates that the lipid packing (i.e., the interactions among the lipid chains) is not significantly perturbed by LL-III and thus suggests that the peptide did not penetrate inside the hydrophobic core of the bilayer but was rather localized at its surface.

**Figure 4:**
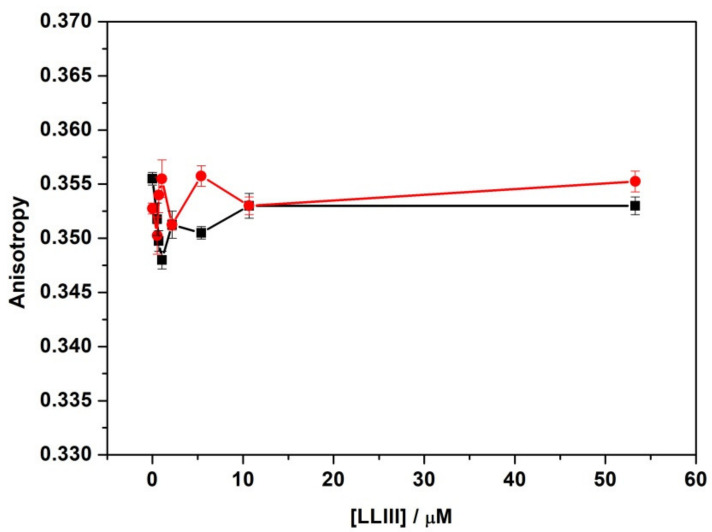
Fluorescence anisotropy of the probe DPH embedded in DPPC (red circles) and in DPPC/DPPG (black squares) as a function of LL-III peptide concentration. The experiments were performed in 10 mM phosphate buffer, pH 7.4, at a temperature of 25 °C.

To further explore the effect of the peptide on lipid packing, fluorescence anisotropy experiments of the probe 1,6-Diphenyl-1,3,5-hexatriene (DPH) were performed. DPH is a fluorescent molecule that partitions inside the hydrophobic interior of the bilayer. Its anisotropy provides information about the lipid packing and how an externally added substance can affect it. Figure 4 shows the anisotropies of DPH embedded in DPPC or DPPC/DPPG as a function of peptide concentration.

Inspection of Figure 4 reveals that LL-III had a minor effect on the anisotropy of DPH embedded in both DPPC and DPPC/DPPG vesicles. These findings clearly support our DSC results, demonstrating that the lipid packing was not significantly modified by peptide binding, and thus the interaction occurred only on the surface of the model membranes.

### 2.4. The Influence of LL-III Binding on the Hydration and Dynamics of the Lipid Head Groups

**Figure 5:**
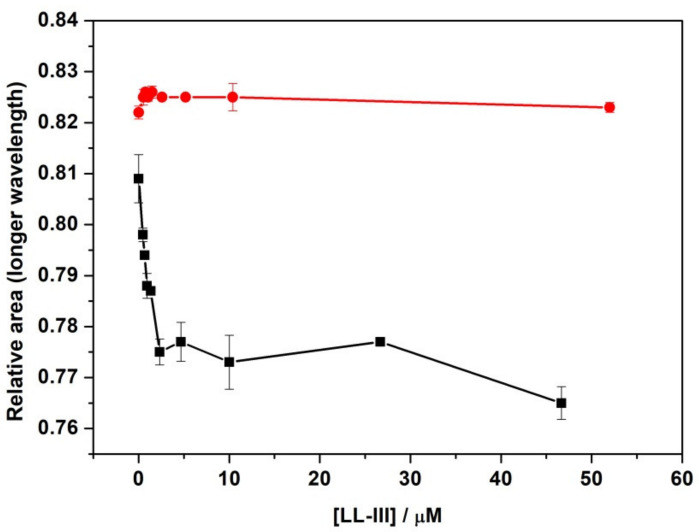
Fractional (or relative) area changes of Laurdan embedded in POPC (red circles) and POPC/POPG (black squares) as a function of LL-III peptide concentration. The experiments were performed in 10 mM phosphate buffer, pH 7.4, at a temperature of 25 °C.

Prompted by the evidence of LL-III surface binding, we decided to monitor closely the changes induced by LL-III binding on the properties of the lipid head group region. Firstly, we monitored the change in the hydration properties of the lipid head groups by fluorescence experiments with a Laurdan probe embedded in POPC and POPC/POPG vesicles. Laurdan is a probe that partitions inside the bilayer with the fluorescent polar-sensitive moiety (naphthalene) mainly localized close to the bilayer/water interface, thus becoming a selective probe of this bilayer region [29,30]. The fluorescence emission spectrum of Laurdan in vesicles shows two emission bands: a lower-wavelength band which is due to emission from an excited state termed non-solvent relaxed state, and a second higher-wavelength band due to emission from the solvent relaxed state. The predominance of the emission from one state with respect to the other one was dependent on the hydration properties of the surface region of the bilayer. It was possible to quantify the extent of hydration of the bilayer surface through deconvolution of the Laurdan emission spectra and by comparing the relative areas attributed to the two states [30]. In Figure 5, the fractional area changes of the longer wavelength bands as a function of peptide concentration for the Laurdan probe embedded in POPC and POPC/POPG vesicles are shown.

The fractional area of the longer wavelength band was defined as the ratio of the area of the band due to the emission from the solvent relaxed state to that of the total area (non-solvent + solvent relaxed states). Thus, a change in this parameter is indicative of a variation of the hydration on the bilayer surface. As shown in Figure 5, for Laurdan incorporated in POPC LUVs, the addition of an increasing concentration of peptide had no effect on the relative area. This result indicates that LL-III was not able to modify at all the hydration state of the surface of the POPC bilayer. Conversely, for POPC/POPG vesicles, a strong change was observed. A significant decrease in the area was detected in the peptide concentration range from 0 to 2.5 μM, pointing out a reduction in the hydration (i.e., water content) in the headgroup region upon peptide binding. These results are consistent with the DSC results revealing major changes in the lateral lipid organization in the DPPC/DPPG but not in the DPPC membrane. These findings were also consistent with the larger Trp fluorescence blue shift value of LL-III recorded in the presence of POPC/POPG compared to the POPC vesicles. Further, this observation suggests that the peptide binding mode to POPC/POPG involves a release of a significant number of water molecules in comparison with the binding to POPC.

**Figure 6:**
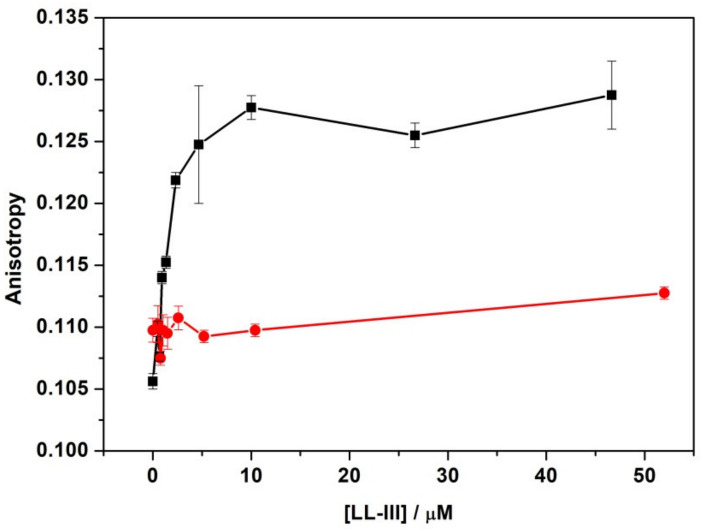
Fluorescence anisotropy of Laurdan embedded in POPC (red circles) and POPC/POPG (black squares) at increasing peptide concentrations. The experiments were performed in 10 mM phosphate buffer, pH 7.4, at a temperature of 25 °C.

Next, we monitored the effect of peptide binding on the dynamic properties of the membrane by measuring the change in the fluorescence anisotropy of Laurdan evaluated at 476 nm where only the solvent relaxed state could contribute [30]. In Figure 6, the anisotropy of the Laurdan probe in POPC and POPC/POPG vesicles at increasing peptide concentrations are shown.

In good agreement with the minor perturbing effect of LL-III on POPC vesicles, the anisotropy of Laurdan in POPC was only marginally affected by the presence of the peptide, even at concentrations higher than 50 μM. Conversely, for POPC/POPG, a significant increase in anisotropy was detected in the same range as for the fractional area reported in Figure 5. These results show that the interaction of the peptide is accompanied by a reduction in the mobility of the probe, indicating that the interfacial dehydration is coupled with a rigidification of the membrane surface.

### 2.5. The Effect of the Peptide on the Membrane Permeability

**Figure 7:**
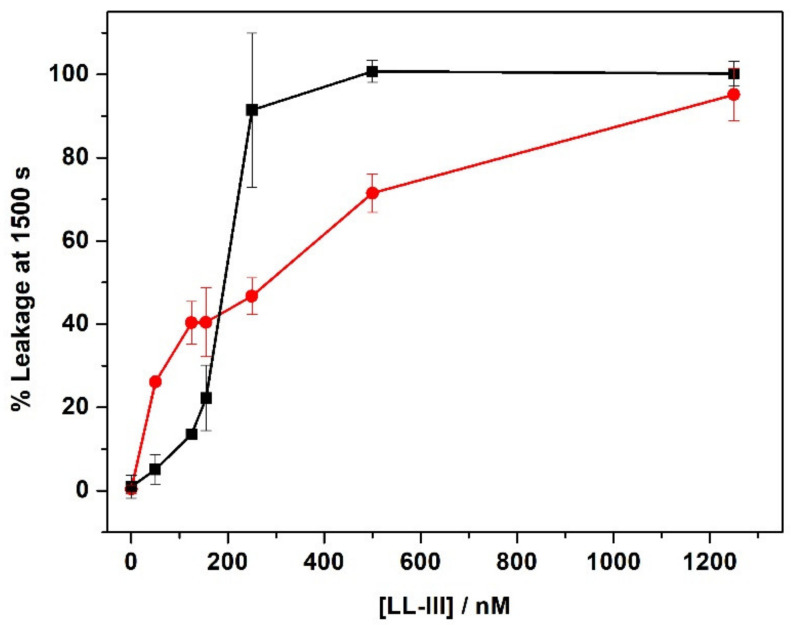
Leakage rate (in %) at t = 1500 s after the peptide addition to LUVs containing the 5-carboxyfluorescein (CF) probe. Red circles: POPC LUVs, black squares: POPC/POPG LUVs. The experiments were performed in 10 mM phosphate buffer, pH 7.4, at a temperature of 25 °C.

Most antimicrobial peptides cause drastic alterations in membrane permeability [31,32]. A common method of quantifying the extent of membrane permeabilization caused by an antimicrobial peptide is via its ability to induce leakage of vesicle-entrapped molecules. For this study, we used 100 nm POPC/POPG and POPC LUVs encapsulating 30 mM 5-carboxyfluorescein (CF), a concentration at which the fluorescence of CF is self-quenched, and we treated the liposomes with the peptide LL-III. A resulting increase in fluorescence due to the release of the dye from liposomes demonstrates that the peptide was able to permeabilize the target membrane. The rate of CF leakage from the vesicles was calculated by the increase in fluorescence emission as a function of time. Figure 7 shows the percentage of leakage obtained for both membranes with increasing peptide concentration. In the absence of peptide, a low fluorescence intensity corresponding to a percentage of 0% leakage was recorded due to the self-quenched and highly concentrated CF entrapped in the inner volume of the LUVs. The addition of LL-III to both membranes caused a fast increase in fluorescence intensity in a concentration-dependent manner, signifying its capability to efficiently permeabilize the membranes. The maximum leakage was observed at a much smaller peptide concentration (~500 nM) for POPC/POPG than for POPC (~1300 nM). Interestingly, the leakage profile of POPC/POPG shows a “sigmoidal” behavior, suggesting a cooperative effect that is likely linked to the initial lipid lateral reorganization of the membrane upon binding.

### 2.6. Peptide Binding to Plasmidic DNA

**Figure 8:**
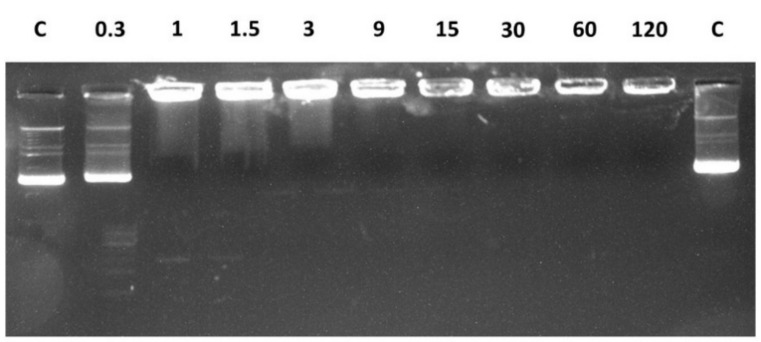
Gel retardation analysis of the binding of LL-III to pUC19 plasmid DNA. Increasing amounts of peptide were incubated with 200 ng of plasmid DNA at room temperature for 30 min. The number above the lanes represents the peptide/DNA weight ratio. Lane C: control, consisting of plasmid DNA only. The image shown is representative of two experiments with the same results.

**Figure 9:**
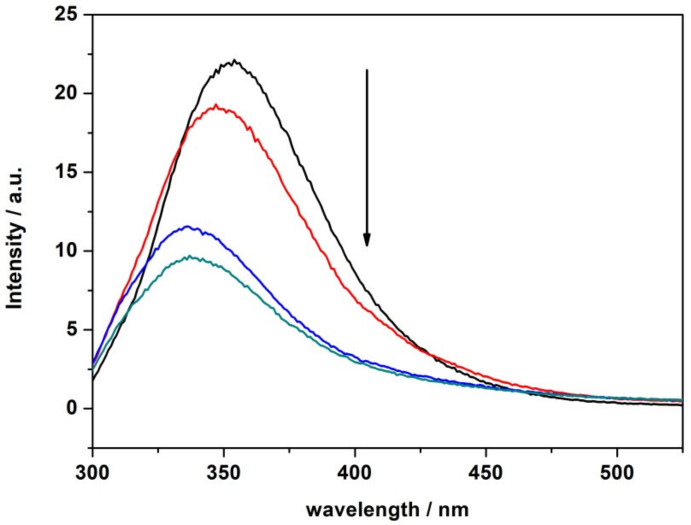
Emission spectra of the free peptide LL-III at the concentration of 4 μM (black line) and after the addition of 0.2 μg (red line), 1.4 μg (blue line), 2.8 μg (green line) of pUC19 plasmid DNA. Black arrow indicates increasing DNA concentration. The experiments were performed in 10 mM phosphate buffer, pH 7.4, at a temperature of 25 °C.

Prompted by the observation that LL-III is able to permeabilize the membrane without disrupting it, we looked for a possible intracellular target of the peptide. To this end, the DNA-interacting ability of LL-III was evaluated by gel retardation assay and fluorescence quenching experiments. Different amounts of the peptide were mixed with a fixed amount (200 ng) of pUC19 plasmid DNA at a peptide/DNA weight ratio 0, 0.3, 1, 1.5, 3, 9, 15, 30, 60, and 120, and the complexes were electrophoresed on a 1% agarose gel (Figure 8). At a peptide/DNA weight ratio of 0.3, a fraction of the plasmid DNA was still able to migrate into the gel in the same way as non-complexed DNA (C). At a weight ratio of 1, significant retardation of the DNA was observed, which increased with increasing amounts of the peptide, clearly demonstrating a DNA/LL-III interaction. The binding of LL-III to DNA was further supported by fluorescence data, where quenching of the Trp fluorescence of LL-III coupled with a strong blue shift of the maximum of emission upon addition of pUC19 DNA was observed (Figure 9).

## 3. Discussion

The antimicrobial peptide LL-III extracted from the venom of the eusocial bee *Lasioglossum laticeps* has shown good antibacterial activity against different strains of Gram-positive and Gram-negative bacteria, anticancer activity against some cancer cells as well as antifungal properties against *Candida albicans* [13,15,16,17]. It was reported that LL-III could target some intracellular components in cancer cells [16]. However, how the peptide might reach the intracellular space is unclear, and the exact mechanism through which its activity is carried out is not well understood. Here, to elucidate the specific role played by the lipid-peptide interactions in the Lasioglossins action mechanism, we performed a comprehensive physico-chemical characterization of the interaction of LL-III with a model of negatively charged pathogenic membranes [19,20,21,22]. Particularly, we employed POPC/POPG liposomes serving as a model for negatively charged pathogenic membranes and liposomes of pure POPC as a simple model of healthy eukaryotic cells.

**Figure A1:**
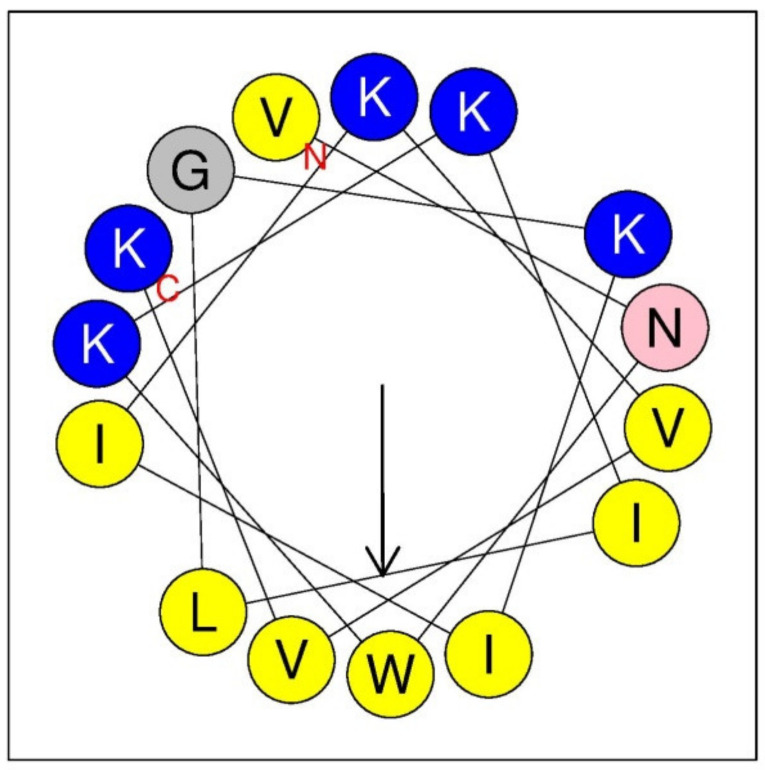
The helical wheel projection of the LL-III peptide. The arrow represents the hydrophobic moment vector (0. 657). Amino acids color code: hydrophobic residues (yellow), positively charged residues (blue), and polar residues with no charge (pink). The projection was made by means of the Heliquest software (http://heliquest.ipmc.cnrs.fr/cgibin/ComputParams.py, accessed date: 05/08/2020).

Firstly, we monitored the ability of the peptide to bind to the lipid bilayers by following the intrinsic fluorescence of the Trp residue located at the N-terminus of the peptide sequence. Our fluorescence titration experiments show that the LL-III binds to POPC and POPC/POPG liposomes, with partition constants (*K_x_*) that are only slightly different. Further, for both membranes, a significant blue shift of the Trp fluorescence maximum was detected, indicating that the Trp residue is well shielded from the aqueous environment. This result was consistent with the well-known preferential localization of the Trp residues at the water/membrane interface through π-cationic interactions [33]. Although this observation could also suggest a penetration of the peptide into the lipid bilayer, DPH anisotropy experiments (Figure 4) unambiguously demonstrate that the peptide does not penetrate into the hydrophobic core of the membrane. DSC experiments further confirm this result showing that the overall enthalpy change of the gel-to-liquid transition is only marginally affected by the presence of the peptide (Table 2), thus revealing that LL-III does not significantly perturb the lipid packing of the two membranes. Overall, our DHP anisotropy and DSC measurements were consistent with the binding of the peptide at the surface level of the bilayer. A deeper analysis of the DSC profile of the DPPC/DPPG vesicles revealed a significant lateral redistribution of the lipid, induced upon peptide binding. This observation could be explained by assuming a selective lipid sorting and binding of the cationic LL-III to the anionic PG lipids. Similar lipid segregation has already been observed for several AMPs, and it is considered a key aspect of their action mechanism [26,34,35,36]. Interestingly, the analysis of the peptide’s CD spectra showed that LL-III adopted a helical conformation in the presence of the POPC/POPG vesicles but remained largely unstructured in the presence of the POPC vesicles, thus suggesting that the α-helix is most likely induced by a selective interaction with the anionic PGs through electrostatic and hydrophobic interactions. This hypothesis is further supported by the observation that the peptide folding into an α-helix leads to the formation of an amphipathic structure (see Figure A1 for the helical wheel projection in the Appendix A section) with a high calculated hydrophobic moment (μ_H_) of 0.657 [37].

Having established that the peptide bound at the surface of lipid bilayers, we then monitored the effect of the peptide on the hydration and dynamics of the lipid head group region of the bilayers, looking for major differences in the two model membranes. To this end, we employed the Laurdan probe known to be selective for the bilayer/water interface region. Analysis of the fluorescence spectra and of the anisotropy measurements of Laurdan probes embedded in POPC and POPC/POPG at increasing LL-III peptide concentrations (Figure 5 and Figure 6) showed that POPC was only marginal affected by the presence of the peptide, whereas binding of the peptide to the POPC/POPG membranes led to dehydration associated with membrane surface stiffening.

Altogether, these results suggest that the surface binding of LLIII with the two membranes happens in a significantly different way. In the case of POPC, the binding to the bilayer/water interface did not result in a major change in the hydration of the membrane. This result can be explained by assuming that the peptide is “anchored” to the surface of the membrane with just a few residues (at least the Trp residue at the N-terminus) but does not lay on the surface for its entire length. This mode of binding could also explain the observation that the Laurdan probe anisotropy is not affected; furthermore, it is also consistent with the disordered conformation of the bound peptide observed by CD. On the contrary, the surface binding of LL-III to the POPC/POPG membrane resulted in a major change in hydration and rigidification of the membrane. This result, together with the observed induced conformational change observed by CD spectroscopy, suggests that the peptide lays on the surface with its entire length, establishing a plethora of membrane-peptide interactions with most of its residues and involving several lipid head groups.

Additionally, our data show that the interaction of the peptide with both membranes takes place in a non-destructive way, supporting a non-lytic action mechanism of LL-III. We then investigated the ability of the peptide to permeabilize the membranes. The leakage experiments performed with CF entrapped in the inner core of the liposomes indicate that LL-III induces permeabilization of both membranes. However, the maximum effect was reached at a much smaller LL-III concentration for the POPC/POPG in comparison with the POPC membrane (according to the low cytotoxicity observed for LL-III). The efflux of an entrapped fluorescent probe was often interpreted as the presence of pores induced by the peptide in the lipid bilayer. However, we found that this process was peptide-concentration dependent, which indicates that no true pores are formed upon binding to the membrane [31]. Interestingly, the leakage profile of POPC/POPG showed a “sigmoidal” behavior, suggesting a cooperative effect that is likely linked to the initial lipid lateral reorganization of the membrane upon binding of the peptide. This hypothesis was consistent with the DSC data (Figure 3), showing that the formation of domains was strictly dependent on the peptide concentration. Indeed, changes in the main DSC transition peak were observable from L/P = 100 (beginning of the main peak) to L/P = 10 (separation of the peak in two distinct peaks). A similar correlation between domain formation and membrane permeabilization has been previously suggested/observed by other authors [10,36]. In addition, the promotion of domain formation has been suggested to favor the translocation of the peptide inside the intracellular space (e.g., via defect states at domain boundaries), where different macromolecules (e.g., proteins and nucleic acids) can be targeted by the peptide [36]. The membrane permeabilization driven by the lipid segregation seems thus a reasonable hypothesis to explain the observed concentration-dependent and cooperative behavior observed in the leakage experiments. This hypothesis could also explain the reported observation that LL-III enters the cells in higher quantities and quickly only after it reaches the toxic concentration [16]. We finally explored the ability of LL-III to bind bacterial DNA by means of gel retardation assay and fluorescence experiments. Both techniques revealed that LL-III was able to bind to bacterial DNA (Figure 8 and Figure 9), suggesting a possible intracellular target for this peptide. On the other hand, the presence of a specific intracellular target in the pathogenic cells and/or the much lower membrane permeabilization ability observed for POPC liposomes could provide the first clue to explain the low toxic effect of LL-III against healthy eukaryotic cells.

In summary, our data clearly indicate that the LL-III peptide is able to interact with anionic model membranes through selective interaction with PGs, which leads to the formation of lipid domains within the lipid bilayer plane. It is noteworthy that the interaction process occurs without any destabilization of the lipid packing, i.e., lipid-lipid interactions are preserved upon peptide binding. Nevertheless, LL-III induces efflux of entrapped CF from the lipid vesicles. The permeabilization occurs most likely through membrane reorganization upon binding. During the rearrangement of lipids molecules, CF may be released, suggesting the possibility that the peptide gains access to the cytosolic side of the cell and attacks intracellular biomolecules such as nucleic acids. Consistently with this hypothesis, our gel-retardation assay experiments showed that LL-III could interact with plasmid DNA, suggesting a possible intracellular target. Overall, our data provide a detailed biophysical characterization of the interaction of LL-III with lipid bilayers, helping to clarify the mechanism of action of the LL-III peptide. This peptide, with its remarkable biological properties, has the potential to serve as an agent for the treatment of bacterial infections as well as to inhibit the growth of tumor cells.

## 4. Materials and Methods

### 4.1. Materials

The peptide Lasioglossin III (sequence: VNWKKILGKIIKVVK-NH_2_) was chemically synthesized and purchased from Primm srl, Milano, Italy. The peptide used had a purity of >95%. The lipids 1-palmitoyl-2-oleoyl-*sn*-glycero-3-phosphocholine (POPC), 1-palmitoyl-2-oleoyl-*sn*-glycero-3-*rac*-phosphoglycerol (POPG), and 1,2-dipalmitoyl-*sn*-glycero-3-phosphoglycerol (DPPG) were obtained from Avanti Polar Lipids Inc. (Alabaster, AL, USA). Meanwhile, the lipid 1,2-dipalmitoyl-*sn*-glycero-3-phosphocholine (DPPC) was purchased from Sigma-Aldrich Chemical. The lipids were used without further purification. The fluorescent membrane probes DPH (1,6-Diphenyl-1,3,5-hexatriene) and Laurdan (6-Dodecanoyl-*N,N*-dimethyl-2-naphthylamine) were obtained from Sigma Aldrich Chemical. Chloroform and methanol were purchased from Sigma Aldrich Chemical. The phosphate buffer, 10 mM at pH 7.4, was prepared using deionized water.

### 4.2. Vesicles Preparation

The lipids were weighted in a glass vial and dissolved in a chloroform/methanol mixture (2/1 *v/v*). A thin film was produced by evaporating the organic solvent with dry nitrogen gas. Lipid film samples were kept under vacuum for at least 4 h to remove the residual traces of the organic solvent. Dry lipids were then hydrated with 10 mM phosphate buffer pH 7.4, and shaken on a vortex mixer, obtaining a suspension of multilamellar vesicles (MLVs). To obtain multilamellar vesicles containing the fluorescent probe DPH (1,6-Diphenyl-1,3,5-hexatriene), the previously described procedure was followed, taking care of adding a solution of DPH in chloroform to the lipid organic mixture at a lipid/DPH mole ratio of 150. The MLVs containing the fluorescent probe Laurdan (6-Dodecanoyl-*N,N*-dimethyl-2-naphthylamine), were instead obtained by adding to the lipids dissolved in the organic mixture a solution of Laurdan in dimethylformamide (DMF) at a lipid/Laurdan mole ratio of 30.

To obtain large unilamellar vesicles (LUVs) of 200 nm size, the MLVs were then extruded at least 21 times through two stacked polycarbonate filters (Nuclepore, Pleasanton, CA, USA) using a mini-extruder (Avanti Polar Lipids Inc., Alabaster, AL, USA) fitted with two 0.25 mL Hamilton syringes (Hamilton, Reno, NV, USA) [38,39]. Their size was confirmed by dynamic light scattering measurements by means of a Zetasizer nano-ZS (Malvern Instruments, Malvern, UK). The mean hydrodynamic radius was consistent with the formation of unilamellar vesicles.

Liposomes with different composition were prepared: (i) DPPC, (ii) POPC, (iii) DPPC/DPPG (8/2 mol/mol); (iv) POPC/POPG (8/2 mol/mol) and (v) DPPC/POPG (8/2 mol/mol). Samples of liposomes in the presence of peptides were prepared by mixing peptide solutions and liposome suspensions to get the desired lipid-to-peptide (L/P) ratio.

### 4.3. Circular Dichroism Spectroscopy (CD)

A Jasco J-715 CD Spectropolarimeter (Jasco Analytical Instruments, Tokyo, Japan) was used to perform the measurements. Spectra were acquired in the wavelength range of 190 to 260 nm, with 0.5 nm step resolution, sweep speed of 20 nm min^−1^, a response time of 4 s, and a bandwidth of 2 nm, using a 0.1 cm path length quartz cuvette, at a temperature of 25 °C. Each experiment was reported as the average of 8 accumulated scans. LL-III samples at a final concentration of 15 μM were prepared in NaP 10 mM pH 7.4 buffer in the absence and in the presence of POPC and POPC/POPG LUVs at a total lipid concentration of 750 μM (L/P ratio of 50). The spectra were processed with JASCO software. Solvent spectral subtraction was performed. The peptide’s secondary structure content was calculated from the CD spectra using “PEPFIT Analyses” software [24,25].

### 4.4. Differential Scanning Calorimetry (DSC)

The heat capacity of the multilamellar vesicles (MLVs) in the absence or in the presence of the peptide LL-III was measured by differential scanning calorimetry (DSC) using a high-sensitivity Nano DSC (TA Instruments, New Castle, DE), equipped with 300 μL twin gold capillary cells, pressurized to 3 atm prior to the scan. MLVs were used since they provide a better resolution of the phase transition peak [40]. The heating rate was 1 °C/min, and samples were scanned from 20 to 55 °C. Repeated heating and cooling scans are routinely performed to verify reversibility and reproducibility. The phosphate buffer was measured separately using the same settings, and the buffer curve was subtracted from the thermograms using Origin Lab Software. Data analysis was performed by means of the Nano-Analyze software package provided by the manufacturer. The measured power was converted to specific heat capacity (ΔC_p_) in kJ mol^−1^ K^−1^. The enthalpy values were obtained by direct integration of the area under the baseline subtracted peaks.

### 4.5. Steady-State Fluorescence Spectroscopy

All the fluorescence experiments were performed using a Fluoromax-4 spectrofluorometer (Horiba, Edison, NJ, USA) operating in the steady-state mode at the temperature of 25 °C.

#### 4.5.1. Binding Experiments

Peptide-phospholipid interactions were studied by monitoring the changes in the Trp fluorescence emission spectra of the peptides upon the addition of LUVs. The titrations were performed by recording the spectra of the peptide solution at a fixed concentration of 7 μM and lipid vesicle concentrations ranging from 0 to 2 mM. Trp fluorescence was measured using a quartz cuvette with a path length of 1 cm and a chamber volume of 2 mL under constant stirring.

Emission spectra were recorded between 300 and 500 nm with an excitation wavelength of 280 nm, at slit widths of 6 nm for excitation and 10 nm for emission. Cross-oriented polarizers (Em_pol_ = 0° and Ex_pol_ = 90°) were used to minimize the scattering background [41]. Fluorescence was normalized to the value in the absence of membranes. The binding curve was obtained by plotting the relative fluorescence intensities (F/F_0_) at 329 nm versus lipid vesicles concentration, where F and F_0_ are fluorescence intensities of the peptide in the presence and in the absence of LUVs, respectively. The experimental data were fitted in order to obtain the mole-fraction partition constant (K_x_), as described previously [26].

The ability of LL-III to interact with plasmid DNA pUC19 was also explored by means of fluorescence spectroscopy. Briefly, emission spectra of a 4 μM solution of LL-III in the absence and presence of 0.2, 1.4, and 2.8 μg of pUC19 were recorded using a 1 cm path length quartz cuvette. The excitation wavelength was set to 280 nm, and emission spectra were recorded in the 300–525 nm range at a temperature of 25 °C. The slits were set to 6 and 10 nm for the excitation and emission monochromators, respectively. The obtained spectra were corrected for the absorbance of DNA at the excitation wavelength [42,43].

#### 4.5.2. Emission Spectra of the Laurdan Probe

Laurdan fluorescence emission spectra were recorded by exciting LUVs labeled with Laurdan probes at 340 nm. Emission spectra of Laurdan probes were recorded between 380 and 600 nm at slit widths of 3 nm for excitation wavelength and 7 nm for emission wavelength. To minimize liposome scattering, the employed final lipid concentration was 50 μM. For POPC and POPC/POPG LUVs, emission spectra of Laurdan probes were recorded by varying the peptide concentration. The spectra for all concentrations were decomposed into two Gaussian bands, using the software Origin Pro 8, and the fraction area of the shorter and the longer wavelength bands was calculated. The fluorescence spectrum was transformed from wavelength to energy, and the intensity was multiplied by λ^2^, considering that the spectrum is recorded with constant wavelength resolution, not energy [30,42]. This methodology has an advantage over the calculation of the Excitation Generalized Polarization by clearly separating the fluorescence emission of the two different excited states of Laurdan.

#### 4.5.3. Fluorescence Anisotropy

Fluorescence anisotropy measurements were carried out for the DPH and Laurdan probes embedded into LUVs at a total lipid concentration of 50 μM. For the DPH probe, the excitation wavelength was set to 355 nm, the emission was monitored at 425 nm, and the slits were set to 8 nm for the excitation monochromator and 12 nm for the emission monochromator. While for the Laurdan probes, the excitation wavelength was set to 340 nm, the emission was monitored at 476 nm, and the slits were set to 9 nm for the excitation monochromator and 14 nm for the emission monochromator. The experiments were performed using a 1 cm path length quartz cuvette with a chamber volume of 150 μL. Fluorescence anisotropies (r) were determined according to the relation: r = (I_VV_ − GI_VH_)/(I_VV_ + 2GI_VH_), where I_VV_ is the fluorescence intensity obtained by setting both the excitation and emission polarizers vertically, I_VH_ is the fluorescence intensity obtained by setting the excitation polarizer vertically and the emission polarizer horizontally, and G is an instrument-specific correction factor [42,43].

### 4.6. Entrapment of CF in LUVs and Leakage Measurements

Perturbation of membrane permeability was determined by measuring the release of a fluorophore entrapped inside liposomes. LUVs composed of POPC/POPG (8/2 mol/mol) and POPC were used. LUVs were prepared by dissolving the required amount of dry lipids in a chloroform/methanol mixture (2/1 *v/v*). The solvent was removed by dry nitrogen gas to form a thin lipid film. After being dried under vacuum at least for 4 h, the lipids were hydrated with 10 mM Tris HCl, pH 7.4, containing 30 mM 5-Carboxyfluorescein (CF). At this concentration, the fluorescence of CF was self-quenched. The suspension was extruded through polycarbonate filters (100 nm pore-size filters, 21 times) by a mini-extruder (Avanti Polar Lipids Inc., Alabaster, AL, USA). Free CF was removed by gel filtration chromatography, passing the extruded LUVs through a Sephadex G25 medium column (1.45 × 5.0 cm) eluted with 50 mM Tris HCl, pH 7.4, containing 150 mM NaCl. This ensured that the inner and the outer vesicle solutions had the same osmolarity. The LUVs containing CF exceed the pore size of the resin and will elute in the void volume of the column. The free CF molecules elute later because they enter the resin beads as they pass through the column. Lipids are lost during preparation, especially during extrusion and gel filtration. For this reason, the lipid content was determined by the Stewart assay [44]. The leakage kinetics were followed by the fluorescence intensity increase due to CF release from LUVs after the addition of LL-III. Entrapped LUVs in suspensions containing 2 μM lipids were incubated with various concentrations of the peptide (0–1.25 μM). The fluorescence of the released CF was recorded continuously with a Horiba Jovin Yvon Fluoromax-4 spectrofluorometer at 25 °C using an excitation wavelength of 493 nm and an emission wavelength of 515 nm. At the end of each experiment, the maximum fluorescence intensity corresponding to 100% leakage was determined by the addition of 10% Triton X-100 (10 μL) to 1 mL of the sample. The percentage of CF leakage caused by the peptide was calculated in accordance with the following equation

Leakage (%) = (I_t_ − I_0_)/(I_T_ − I_0_) × 100 (1)

where I_t_ is the fluorescence intensity after addition of peptide at time t = 1500 s, I_0_ represents the fluorescence of the intact vesicles, and I_T_ is the fluorescence intensity after the addition of the detergent Triton X-100.

## Figures and Tables

**Table 1:** Percentages of secondary structure elements obtained from the deconvolution of CD spectra of LL-III peptide in the absence and in the presence of the reported LUVs at a lipid-to-peptide ratio (L/P) = 50.

System	α-Helix	β-Turn ^1^	Random Coil	R^2^
LL-III in buffer	15%	23%	62%	0.982
+POPC	20%	29%	51%	0.986
+POPC/POPG	89%	11%	-	0.990

^1^ Sum of type I, II, and III β-turns.

**Table 2:** Thermodynamic parameters for the phase transitions of MLVs of DPPC and DPPC/DPPG in the absence and presence of LL-III peptide at the indicated lipid-to-peptide ratios.

System	^1^ T_p_/°C	^1,3^ ΔH_p_/kJ mol^−1^	^2^ T_m_/°C	^2,3^ ΔH_m_/kJ mol^−1^
DPPC	33.4	2.1	40.9	34.1
L/P = 50	32.2	2.0	40.9	37.5
L/P = 10	31.5	1.2	40.9	35.5
DPPC/DPPG	33.3	1.9	41.0	36.0
L/P = 100	33.4	0.8	41.6	37.5
L/P = 50	-	-	42.2	42.3
L/P = 10	-	-	42.1	36.2

^1^ Temperature and enthalpy change for the pre-transition; ^2^ Temperature and enthalpy change for the main transition; ^3^ Normalization by the total lipid concentration. The errors in T_p_ are ± 0.2 °C, and errors in T_m_ are ± 0.1 °C. Errors on ΔH are ± 5% of the reported values.

## Data Availability

The data presented in this study are available on request from the corresponding author.

## Abbreviations

AMPs	Antimicrobial peptides
CD	Circular Dichroism
CF	5-Carboxyfluorescein
DPPC	1,2-dipalmitoyl-sn-glycero-3-phosphocholine
DPPG	1,2-dipalmitoyl-sn-glycero-3-phosphoglycerol
DPH	1,6-Diphenyl-1,3,5-hexatriene
DSC	Differential Scanning Calorimetry
L/P	Lipid-to-peptide mole ratio
LUVs	Large unilamellar vesicles
MLVs	Multilamellar vesicles
POPC	1-palmitoyl-2-oleoyl-sn-glycero-3-phosphocholine
POPG	1-palmitoyl-2-oleoyl-sn-glycero-3-rac-phosphoglycerol

## References

[1] Wang G. (2012). Post-Translational Modifications of Natural Antimicrobial Peptides and Strategies for Peptide Engineering. Curr. Biotechnol. E.

[2] Zasloff M., Matsuzaki K. (2019). Antimicrobial Peptides of Multicellular Organisms: My Perspective. Antimicrobial Peptides.

[3] Fan L., Sun J., Zhou M., Zhou J., Lao X., Zheng H., Xu H. (2016). DRAMP: A Comprehensive Data Repository of Antimicrobial Peptides. Sci. Rep..

[4] Wang X., Wang G. (2016). Insights into Antimicrobial Peptides from Spiders and Scorpions. Protein Pept. Lett..

[5] Lei J., Sun L., Huang S., Zhu C., Li P., He J., Mackey V., Coy D.H., He Q. (2019). The Antimicrobial Peptides and Their Potential Clinical Applications. Am. J. Transl. Res..

[6] Hancock R.E.W., Sahl H.-G. (2006). Antimicrobial and Host-Defense Peptides as New Anti-Infective Therapeutic Strategies. Nat. Biotechnol..

[7] Wang G., Li X., Wang Z. (2016). APD3: The Antimicrobial Peptide Database as a Tool for Research and Education. Nucleic Acids Res..

[8] Zasloff M. (2002). Antimicrobial Peptides of Multicellular Organisms. Nature.

[9] Toke O. (2005). Antimicrobial Peptides: New Candidates in the Fight against Bacterial Infections. Biopolymers.

[10] Matsuzaki K. (2019). Antimicrobial Peptides: Basics for Clinical Application.

[11] Brogden K.A. (2005). Antimicrobial Peptides: Pore Formers or Metabolic Inhibitors in Bacteria?. Nat. Rev. Microbiol..

[12] Park C.B., Yi K.-S., Matsuzaki K., Kim M.S., Kim S.C. (2000). Structure-Activity Analysis of Buforin II, a Histone H2A-Derived Antimicrobial Peptide: The Proline Hinge Is Responsible for the Cell-Penetrating Ability of Buforin II. Proc. Natl. Acad. Sci. USA.

[13] Čeřovský V., Buděšínský M., Hovorka O., Cvačka J., Voburka Z., Slaninová J., Borovičková L., Fučík V., Bednárová L., Votruba I. (2009). Lasioglossins: Three Novel Antimicrobial Peptides from the Venom of the Eusocial Bee *Lasioglossum Laticeps* (Hymenoptera: Halictidae). ChemBioChem.

[14] Bandyopadhyay S., Lee M., Sivaraman J., Chatterjee C. (2013). Model Membrane Interaction and DNA-Binding of Antimicrobial Peptide Lasioglossin II Derived from Bee Venom. Biochem. Biophys. Res. Commun..

[15] Vrablikova A., Czernekova L., Cahlikova R., Novy Z., Petrik M., Imran S., Novak Z., Krupka M., Cerovsky V., Turanek J. (2017). Lasioglossins LLIII Affect the Morphogenesis of *Candida Albicans* and Reduces the Duration of Experimental Vaginal Candidiasis in Mice: Lasioglossins in *Candida* Infection. Microbiol. Immunol..

[16] Slaninová J., Mlsová V., Kroupová H., Alán L., Tůmová T., Monincová L., Borovičková L., Fučík V., Čeřovský V. (2012). Toxicity Study of Antimicrobial Peptides from Wild Bee Venom and Their Analogs toward Mammalian Normal and Cancer Cells. Peptides.

[17] Slaninová J., Putnová H., Borovičková L., Šácha P., Čeřovský V., Monincová L., Fučík V. (2011). The Antifungal Effect of Peptides from Hymenoptera Venom and Their Analogs. Open Life Sci..

[18] Oliva R., Mukherjee S.K., Fetahaj Z., Möbitz S., Winter R. (2020). Perturbation of Liquid Droplets of P-Granule Protein LAF-1 by the Antimicrobial Peptide LL-III. Chem. Commun..

[19] Teixeira V., Feio M.J., Bastos M. (2012). Role of Lipids in the Interaction of Antimicrobial Peptides with Membranes. Prog. Lipid Res..

[20] Stillwell W. (2013). An Introduction to Biological Membranes From Bilayers to Rafts.

[21] Travkova O.G., Moehwald H., Brezesinski G. (2017). The Interaction of Antimicrobial Peptides with Membranes. Adv. Colloid Interface Sci..

[22] Ran S., Downes A., Thorpe P.E. (2002). Increased Exposure of Anionic Phospholipids on the Surface of Tumor Blood Vessels. Cancer Res..

[23] Oliva R., Del Vecchio P., Stellato M.I., D’Ursi A.M., D’Errico G., Paduano L., Petraccone L. (2015). A Thermodynamic Signature of Lipid Segregation in Biomembranes Induced by a Short Peptide Derived from Glycoprotein Gp36 of Feline Immunodeficiency Virus. Biochim. Biophys. Acta BBA Biomembr..

[24] Reed J., Reed T.A. (1997). A Set of Constructed Type Spectra for the Practical Estimation of Peptide Secondary Structure from Circular Dichroism. Anal. Biochem..

[25] Amon M.A., Ali M., Bender V., Hall K., Aguilar M.-I., Aldrich-Wright J., Manolios N. (2008). Kinetic and Conformational Properties of a Novel T-Cell Antigen Receptor Transmembrane Peptide in Model Membranes. J. Pept. Sci..

[26] Oliva R., Del Vecchio P., Grimaldi A., Notomista E., Cafaro V., Pane K., Schuabb V., Winter R., Petraccone L. (2019). Membrane Disintegration by the Antimicrobial Peptide (P)GKY20: Lipid Segregation and Domain Formation. Phys. Chem. Chem. Phys..

[27] Pizzo E., Oliva R., Morra R., Bosso A., Ragucci S., Petraccone L., Del Vecchio P., Di Maro A. (2017). Binding of a Type 1 RIP and of Its Chimeric Variant to Phospholipid Bilayers: Evidence for a Link between Cytotoxicity and Protein/Membrane Interactions. Biochim. Biophys. Acta BBA Biomembr..

[28] Oliva R., Chino M., Lombardi A., Nastri F., Notomista E., Petraccone L., Del Vecchio P. (2020). Similarities and Differences for Membranotropic Action of Three Unnatural Antimicrobial Peptides. J. Pept. Sci..

[29] Gironi B., Oliva R., Petraccone L., Paolantoni M., Morresi A., Del Vecchio P., Sassi P. (2019). Solvation Properties of Raft-like Model Membranes. Biochim. Biophys. Acta BBA Biomembr..

[30] Lúcio A.D., Vequi-Suplicy C.C., Fernandez R.M., Lamy M.T. (2010). Laurdan Spectrum Decomposition as a Tool for the Analysis of Surface Bilayer Structure and Polarity: A Study with DMPG, Peptides and Cholesterol. J. Fluoresc..

[31] Wimley W.C. (2010). Describing the Mechanism of Antimicrobial Peptide Action with the Interfacial Activity Model. ACS Chem. Biol..

[32] Wimley W.C., Hristova K. (2020). The Mechanism of Membrane Permeabilization by Peptides: Still an Enigma. Aust. J. Chem..

[33] Yau W.-M., Wimley W.C., Gawrisch K., White S.H. (1998). The Preference of Tryptophan for Membrane Interfaces. Biochemistry.

[34] Phambu N., Almarwani B., Alwadai A., Phambu E.N., Faciane N., Marion C., Sunda-Meya A. (2017). Calorimetric and Spectroscopic Studies of the Effects of the Cell Penetrating Peptide Pep-1 and the Antimicrobial Peptide Combi-2 on Vesicles Mimicking *Escherichia Coli* Membrane. Langmuir.

[35] Wadhwani P., Epand R.F., Heidenreich N., Bürck J., Ulrich A.S., Epand R.M. (2012). Membrane-Active Peptides and the Clustering of Anionic Lipids. Biophys. J..

[36] Epand R.F., Maloy W.L., Ramamoorthy A., Epand R.M. (2010). Probing the “Charge Cluster Mechanism” in Amphipathic Helical Cationic Antimicrobial Peptides. Biochemistry.

[37] Gautier R., Douguet D., Antonny B., Drin G. (2008). HELIQUEST: A Web Server to Screen Sequences with Specific-Helical Properties. Bioinformatics.

[38] Hope M.J., Bally M.B., Webb G., Cullis P.R. (1985). Production of Large Unilamellar Vesicles by a Rapid Extrusion Procedure. Characterization of Size Distribution, Trapped Volume and Ability to Maintain a Membrane Potential. Biochim. Biophys. Acta BBA Biomembr..

[39] MacDonald R.C., MacDonald R.I., Menco B.P.M., Takeshita K., Subbarao N.K., Hu L. (1991). Small-Volume Extrusion Apparatus for Preparation of Large, Unilamellar Vesicles. Biochim. Biophys. Acta BBA Biomembr..

[40] Biltonen R.L., Lichtenberg D. (1993). The Use of Differential Scanning Calorimetry as a Tool to Characterize Liposome Preparations. Chem. Phys. Lipids.

[41] Ladokhin A.S., Jayasinghe S., White S.H. (2000). How to Measure and Analyze Tryptophan Fluorescence in Membranes Properly, and Why Bother?. Anal. Biochem..

[42] Lakowicz J.R. (2006). Principles of Fluorescence Spectroscopy.

[43] Oliva R., Chino M., Pane K., Pistorio V., De Santis A., Pizzo E., D’Errico G., Pavone V., Lombardi A., Del Vecchio P. (2018). Exploring the Role of Unnatural Amino Acids in Antimicrobial Peptides. Sci. Rep..

[44] Stewart J.C.M. (1980). Colorimetric Determination of Phospholipids with Ammonium Ferrothiocyanate. Anal. Biochem..

